# Multimodality imaging in the diagnostic approach to a patient with carcinoid heart disease involving four heart valves

**DOI:** 10.1002/ccr3.6152

**Published:** 2022-07-25

**Authors:** Shirin Habibi Khorasani, Mozhgan Parsaee, Niloufar Samiei, Mahshid Hesami, Feridoun Noohi, Saeid Hosseini, Golnaz Houshmand

**Affiliations:** ^1^ Adult Echocardiography Department Rajaie Cardiovascular Medical and Research Center Tehran Iran; ^2^ Echocardiography Research Center Rajaie Cardiovascular Medical and Research Center Tehran Iran; ^3^ Heart Valve Disease Research Center Rajaie Cardiovascular Medical and Research Center Tehran Iran; ^4^ Rajaie Cardiovascular Medical and Research Center Tehran Iran; ^5^ Cardiovascular Intervention Research Center Rajaie Cardiovascular Medical and Research Center Tehran Iran; ^6^ Rajaie Cardiovascular Medical & Research Center Tehran Iran

**Keywords:** cardiac magnetic resonance imaging, cardiac surgery, echocardiography

## Abstract

Carcinoid heart disease is a rare condition that occurs in less than half of patients with carcinoid syndrome. The disease mainly affects right‐sided heart valves; however, in 5%–10%, it can also involve left‐sided valves. This case illustrates the most complicated form of the disease involving four heart valves.

## Graphical abstract

The use of multimodality imaging in diagnosing and managing patients with systemic diseases involving the heart is of great importance and may improve the patients' survival and quality of life.

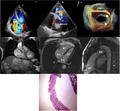

## 1 INTRODUCTION

Carcinoid syndrome represents symptoms caused by neuroendocrine tumors—mainly in the gut—resulting in cardiac involvement in about 20% of patients. The disease usually affects the right heart, and the left heart remains spared, as the lungs metabolize the vasoactive substances causing the disease. However, several conditions may lead to both right‐ and left‐sided involvement. These conditions include primary lung neoplasms, any intracardiac shunts, that is, atrial septal defect (ASD), patent foramen ovale (PFO), etc. and the presence of large burden of neoplastic tissue overwhelming the lung inactivation capacity by releasing notable amount of vasoactive substances.^1^

Transthoracic echocardiography (TTE) is the first imaging modality used in most cardiac patients. Screening for heart disease in those patients with carcinoid syndrome is of great importance since the patient may have no significant signs or symptoms in the early stages. Based on current evidence, TTE is the recommended modality to screen and monitor carcinoid heart disease.^2^

Cardiovascular magnetic resonance imaging (CMR) has excellent benefits in the diagnosis and monitoring of carcinoid heart disease. As the involvement of the pulmonary valve may be underestimated by echocardiography, CMR may have a leading role. Furthermore, the cardiac chamber sizes, the degree of valvular regurgitation and the right ventricular function are best appreciated in CMR. Magnetic resonance imaging (MRI) also has the unique capacity of tissue characterization technique, which may aid in the diagnosis of fibrosis and cardiac metastases.^1^, ^2^

Cardiac CT (computed tomography) is rarely used in patients with carcinoid heart disease, and its central role remains to evaluate the coronary arteries and their relationship with myocardial metastases.^3^

Nuclear imaging for routine assessment of carcinoid heart disease is not established; even though PET (Positron emission tomography)/CT has a significant role in detecting cardiac metastases.^2^

The aim of this report was to point out the importance and the necessity of multimodality imaging and multidisciplinary approach in patients with carcinoid heart disease, especially complicated cases.

## 2 CASE PRESENTATION

We describe a 56‐year‐old man who presented with lower extremity edema. He mentioned paroxysmal flushing, episodes of diarrhea accompanied by significant weight loss (24 kg in 18 months) during the past 5 years. He was managed with the impression of inflammatory bowel disease, irritable bowel syndrome and chronic malabsorption before referral to our center without any significant improvement. A cardiology consult was requested to evaluate right‐sided heart disease after developing exertional dyspnea (NYHA class III), abdominal distension, and lower extremity edema within the past 6 months.

He had a blood pressure of 130/70 mmHg, respiratory rate of 20 breaths/min, heart rate of 80 beats/min, temperature of 37°C (axillary), and O_2_ saturation of 95% in room air. His physical examination on presentation was significant for right‐sided S3, holosystolic murmur grade III/VI over the right lower sternal border, and an early diastolic decrescendo murmur grade III/VI over the left upper sternal border. He also had elevated jugular venous pulse (JVP), ascites and 2+ pitting edema in both lower extremities.

### 2.1 Past medical history

His past medical history was significant for medically controlled hypertension and type II diabetes mellitus on Metformin.

### 2.2 Differential diagnosis

Lower extremity edema accompanied with ascites are suggestive of right‐sided heart failure, liver cirrhosis, or portal hypertension. In this scenario, it is necessary to acknowledge the cause of right ventricular failure. The patient's physical examination raised the possibility of secondary failure due to valvular involvement. Besides, the patient had no history in favor of pulmonary thromboembolism or acute right ventricular myocardial infarction. Another item to consider is the cause of valvular involvement, which ranges from trauma, degenerative changes, rheumatic heart disease, tumor involvement, etc., to iatrogenic causes. A thorough history and comprehensive cardiac imaging including echocardiography and MR imaging, would lead to the diagnosis.

### 2.3 Investigations

**FIGURE 1:**
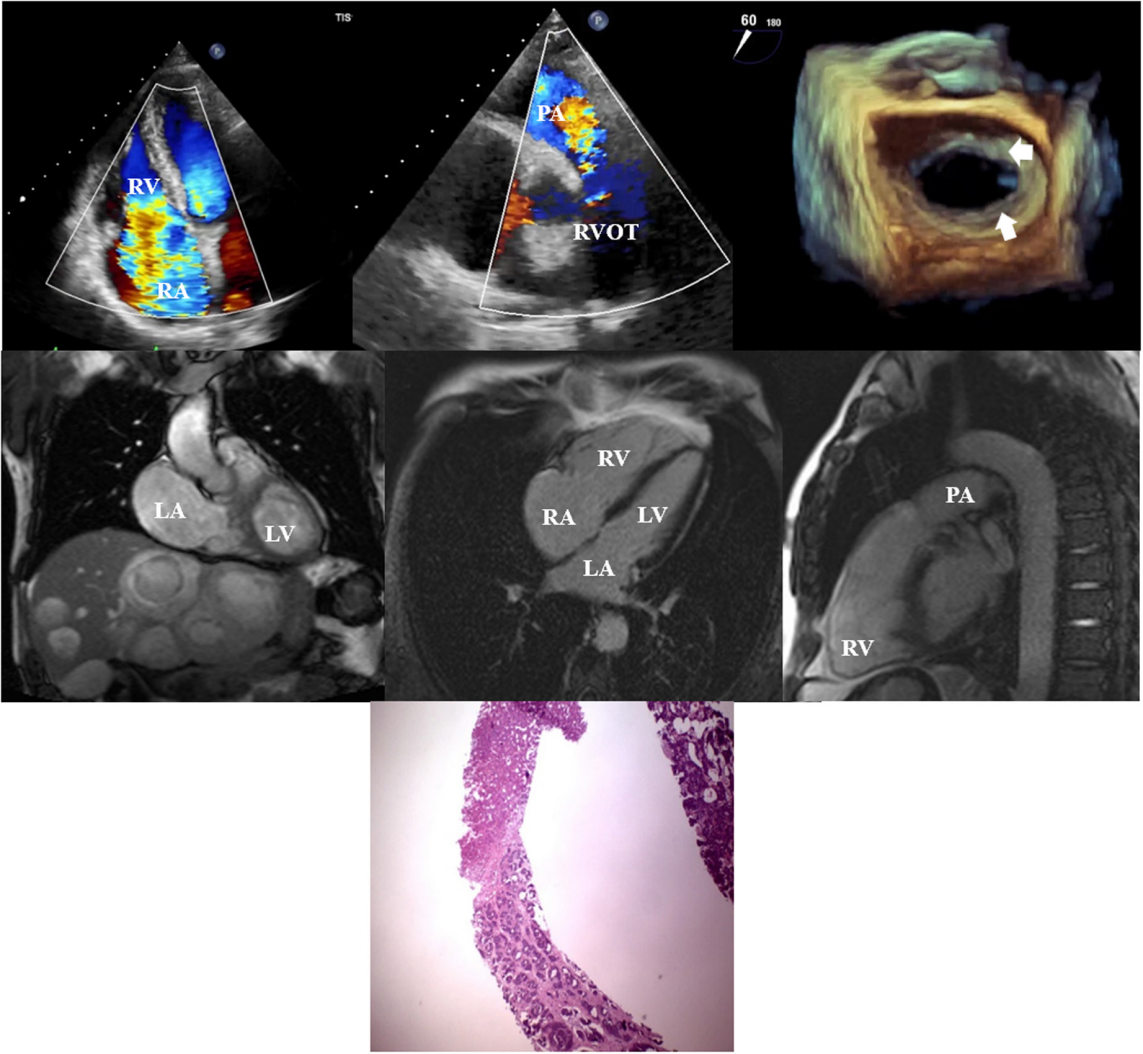
Echocardiographic, CMR, and pathologic findings. (A) Transthoracic echocardiography in apical 4‐chamber view showing non‐coapting tricuspid valve resulting in severe regurgitation. (B) Transthoracic echocardiography in parasternal short‐axis view showing severe pulmonary regurgitation due to involved pulmonary valve. (C) 3D‐reconstruction of mitral valve from transesophageal echocardiography depicting retracted and thickened leaflets. (D) Cardiac Magnetic Resonance (CMR) morphology sequences demonstrating multiple hepatic masses. (E and F) Late gadolinium enhancement (LGE) images showing no myocardial fibrosis but fibrosis in the pulmonary valve annulus. (G) Hematoxylin and Eosin‐stained sections are revealing liver tissue involved by neoplasm composed of solid nests of tumoral cells suggestive of well‐differentiated neuroendocrine (carcinoid) tumor.

The transthoracic echocardiography (TTE) showed normal left ventricular systolic function, moderate to severely enlarged and mildly dysfunctional right ventricle. Thickened aortic and mitral valve leaflets with moderate to severe regurgitation were noted. Both tricuspid and pulmonic valves were thickened, immobile and retracted with a “drum‐stick” appearance resulting in very severe regurgitations (Figure 1A,B, Video 1). The patient had mild pulmonary hypertension (systolic pulmonary artery pressure = 40–45 mmHg) and plethoric inferior vena cava (IVC). Agitated saline contrast administration did not show any bubble passage through interatrial septum with the Valsalva maneuver (Video 1). Transesophageal echocardiography confirmed TTE data (Figure 1C, Video 2). Cardiac Magnetic Resonance (CMR) morphology sequences showed multiple hepatic masses (Figure 1D). Cine sequences showed severely enlarged right ventricle (146 ml/m^2^) with normal ejection fraction (Video 3). Volumetric and flow velocity assessment confirmed torrential tricuspid regurgitation (TR) with a regurgitant fraction (RF) of 65%, severe pulmonary regurgitation (RF = 43%), severe aortic regurgitation (RF = 41%), and moderate mitral regurgitation (RF = 27%). In the late gadolinium enhancement images, there was no myocardial fibrosis; however, fibrosis in the pulmonary valve annulus was noted (Figure 1E,F). Color Doppler sonography of the hepatic artery and portal venous system showed no evidence of portal hypertension or thrombosis. Several laboratory examinations were performed regarding the incidental discovery of hepatic masses and the aforementioned findings. Laboratory results showed normocytic, normochromic anemia (Hemoglobin = 12 mg/dl), elevated Chromogranin‐A level (834 ng/ml, normal value below 100 ng/ml), elevated liver enzymes and normal renal function tests. Liver biopsy confirmed well‐differentiated neuroendocrine (carcinoid) tumor and no evidence of cirrhosis. Hematoxylin and Eosin‐stained sections show liver tissue involved by neoplasm composed of solid nests of tumoral cells (Figure 1G).

### 2.4 Management

Octreotide, diuretics, and angiotensin receptor blockers were prescribed. However, despite the improvement of carcinoid syndrome symptoms, the right‐sided heart failure symptoms persisted. After multidisciplinary heart team discussion and regarding the pre‐operative assessment with EuroSCORE of 2.09%,^4^ four‐valve replacement surgery was recommended for the patient. The surgery was carried out with implantation of mitral (Medtronic Mosaic bioprosthesis no 31), aortic (Carpentier‐Edwards PERIMOUNT bioprosthesis no 23), pulmonic (Carpentier‐Edwards Magna bioprosthesis no 25), and tricuspid (Hancock bioprosthesis no 33) prostheses. The patient recovered favorably following surgery and was discharged in a good state of health.

### 2.5 Follow‐Up

At the most recent clinical visit (6 months after the surgery), he had improved functional capacity and regained much of his lost body weight. In the follow‐up echocardiography, all four valves were optimally functional without complications.

## 3 DISCUSSION

Carcinoid heart disease is a rare condition and occurs in nearly half of patients with carcinoid syndrome and might be the initial manifestation of this syndrome in 20% of patients.^5^ In this condition, a large amount of circulating serotonin (5‐hydroxytryptamine) secreted by hepatic metastases of the carcinoid tumor reaches the right heart and leads to fibrous endocardial depositions in the tricuspid and pulmonary valves.^6^

The vasoactive substances secreted by the metastatic carcinoid tumor are usually metabolized and deactivated in the pulmonary circulation. Therefore, the substances that reach left heat are usually deactivated. Left‐sided heart valve involvement is infrequent and can occur in about 5%–10% of patients in certain circumstances, including intracardiac shunts (i.e., ASD, PFO), bronchial carcinoid tumors and a high burden of tumoral mass.^7^ In our patient, the lack of bubble passage through IAS with the Valsalva maneuver made the left side involvement more attributable to extensive liver metastases. Beyond the systemic presentations, carcinoid heart disease is a catastrophic presentation of carcinoid syndrome with significant morbidity and median survival of fewer than 5 years. Previous studies have mentioned that the median survival depends on timely diagnosis and management of patients, which requires multimodality cardiovascular imaging including echocardiography, CMR, CT, and PET.^8^

In literature, there are reports of carcinoid heart disease involving the left and right heart, which were managed by tumor debulking and repair/replacement of the affected valves in certain cases.^9^ We followed the same approach; however, regarding the oncologist consult, tumor debulking was not recommended for our patient.

In a long‐term follow‐up study of 240 patients in the Mayo Clinic, valve replacement surgery revealed acceptable short‐term mortality. Besides, the earlier the intervention made, the more improved survival was noted.^10^ In our patient, valve replacement along with medical treatment showed satisfying results in short‐term.

## 4 CONCLUSIONS

In patients with multivalvular involvement, a multidisciplinary approach and thorough imaging assessment may aid in early diagnosis and guide necessary cardiac interventions. Thus, the patients' survival and quality of life can be improved.

## AUTHOR CONTRIBUTIONS

SHK had contact with the patient and wrote the manuscript. MP involved in patient's diagnosis and image analysis. NS involved in patient's management and manuscript preparation. MH performed pathological analysis and was involved in manuscript preparation. FN involved in data requisition and manuscript preparation. SH performed surgery and co‐wrote the manuscript. GH conceptualized the case and was involved in all the writing steps.

## CONFLICT OF INTEREST

Nothing to Disclose.

## CONSENT

Written informed consent was obtained from the patient to publish this report in accordance with the journal‘s patient consent policy.

## Supporting information


Video 1

Video 2

Video 3

## Data Availability

Data sharing is not applicable to this article as no new data were created or analyzed in this study.
